# Policy, toxicology and physicochemical considerations on the inhalation of high concentrations of food flavour

**DOI:** 10.1038/s41538-020-00075-y

**Published:** 2020-10-07

**Authors:** Vlad Dinu, Azad Kilic, Qingqi Wang, Charfedinne Ayed, Abdulmannan Fadel, Stephen E. Harding, Gleb E. Yakubov, Ian D. Fisk

**Affiliations:** 1 National Centre for Macromolecular Hydrodynamics, School of Biosciences, University of Nottingham, Sutton Bonington Campus, Leicestershire, UK; 2 Division of Food Sciences, School of Biosciences, University of Nottingham, Sutton Bonington Campus, Leicestershire, UK; 3 Plant and Crop Sciences, School of Biosciences, University of Nottingham, Sutton Bonington Campus, Leicestershire, UK; 4 Centre for Plant Integrative Biology (CPIB), School of Biosciences, University of Nottingham, Sutton Bonington Campus, Leicestershire, UK; 5 School of Biosciences, University of Nottingham, Sutton Bonington Campus, Leicestershire, UK; 6 Sport and Exercise Sciences, Liverpool John Moores University, Byrom Street, Liverpool, UK

**Keywords:** Drug regulation, Chemical safety, Environmental impact, Toxicology

## Abstract

Food flavour ingredients are required by law to obtain prior approval from regulatory bodies, such as the U.S. Food and Drug Administration (FDA) or the European Food Safety Authority (EFSA) in terms of toxicological data and intended use levels. However, there are no regulations for labelling the type and concentration of flavour additives on the product, primarily due to their low concentration in food and generally recognised as safe (GRAS) status determined by the flavour and extract manufacturers’ association (FEMA). Their status for use in e-cigarettes and other vaping products challenges these fundamental assumptions, because their concentration can be over ten-thousand times higher than in food, and the method of administration is through inhalation, which is currently not evaluated by the FEMA expert panel. This work provides a review of some common flavour ingredients used in food and vaping products, their product concentrations, inhalation toxicity and aroma interactions reported with different biological substrates. We have identified several studies, which suggest that the high concentrations of flavour through inhalation may pose a serious health threat, especially in terms of their cytotoxicity. As a result of the wide range of possible protein-aroma interactions reported in our diet and metabolism, including links to several non-communicable diseases, we suggest that it is instrumental to update current flavour- labelling regulations, and support new strategies of understanding the effects of flavour uptake on the digestive and respiratory systems, in order to prevent the onset of future non-communicable diseases.

## Introduction to flavour: aroma compounds

From a chemical perspective, aroma compounds are low-molecular-weight volatile molecules (typically less than 300 g/mol) capable of stimulating the odour receptors in the nose^1–4^. As a result of their size, they physically diffuse through various matrices into the air and then into the oral and nasal fluids in order to reach the olfactory epithelium^3,5^. From a biological and biochemical perspective, natural odours are constantly being produced by living organisms, i.e., bacteria, plants and animals, throughout their life cycle through the metabolic breakdown of more complex molecules such as proteins, lipids, polyphenols and carbohydrate^6–8^. In our evolution, the sense of smell served as a tool in assisting the essential processes of life: nutrition, excretion and reproduction, such as plants attracting insects and animals to aid with pollination^9,10^. Over 7000 aroma compounds have been identified until 2014^3^. The vast spectrum of animal, plant and fruit compounds arises from diverse environmental conditions and competition amongst plants and animals, in order to ensure their survival, i.e., detecting food, mates or avoiding predators. In the modern human society, the identification of desirable and unpleasant smells has also been crucial in the development of food, pharmaceuticals, cosmetics and other products available on the market. The increasing consumer demand for flavour-rich foods, drinks, perfumes, cosmetics and household products has led to a remarkable development in the chemical and biotechnological processes used in the production of aroma compounds for use as flavourings, in order to enhance the consumer acceptability of various products^11^.

According to the World Health Organisation (WHO), the majority of all deaths are due to non-communicable diseases (NCDs), such as respiratory diseases, cardiovascular diseases, obesity, diabetes and cancers, most of which are caused by the excess of sugar, fat and salt in our diet^12,13^. With the increased awareness to adopt healthier lifestyles, particular areas that have benefited from a constant use and development of flavours are low-calorie foods, vegan and vegetarian diets, cleaning products as well as holistic practices, such as aromatherapy. However, one particular application that has expanded like no other in the past decade is the development of electronic nicotine delivery systems and electronic non-nicotine delivery systems (ENDS/ENNDS), commonly referred to as vapes or e-cigarettes^14^. These products have in principle been designed as a smoking replacement therapy in order to reduce the detrimental complications associated with conventional cigarette smoking. They rapidly turned into a profitable business, and today there are thousands of ENDS and ENNDS (flavour-only) products available to consumers under attractive and unique names like “banana nut bread, cotton candy, watermelon wave, love potion and unicorn puke”^15^. Most of the vaping products now contain very high concentrations of flavouring aroma compounds, with up to a million times higher than originally found in nature. As a result of their direct inhalation, flavour compounds are much more efficiently delivered into the lungs, along with the PG and/or VG solvents in which they are dispersed.

**Fig. 1:**
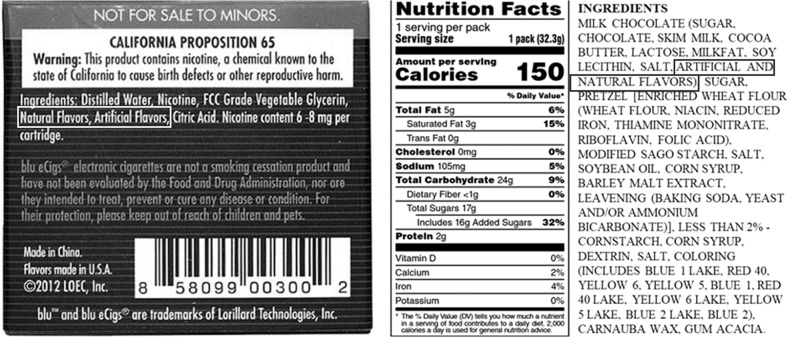
Back-of-pack ingredients and nutrition labels. Examples of ingredient and nutritional information labelled on blu eCigs® flavoured e-cigarettes (left) and on a packet of chocolate candies (right) highlighting the information regarding the type and concentration of flavour used in the formulation. The only available information is generally given as “artificial and/or natural flavours”. The left image is free to be copied and redistributed under the Creative Commons Attribution 2.0 Generic license (cc-by-2.0), and was first published by Lindsay Fox in 2013 on Flickr (https://www.flickr.com/photos/87735223@N02/9832539844).

In foods, the labelling of ingredients along with their nutritional information has served as a prominent intervention strategy for reducing the onset of NCDs by providing consumers and health professionals with the information needed to inspire a healthy and balanced diet. Initiated in the United States in 1990 by the Nutrition Labelling and Education Act, the display of nutritional information on the levels and types of fat, protein, carbohydrate, sugar, salt, vitamins and other essential constituents has become mandatory in almost every country^16^. From June 2018, the Food and Drug Administration (FDA) maintains a publicly available inventory on “Substances Added to Food”; however, current regulations do not require food and cosmetic manufacturers to list the levels and type of flavouring used in a product^17–20^ (Fig. 1). However, the FDA has identified the flavours used in e-cigarettes as a likely target of regulation and began to solicit information regarding the use of flavours. As of January 2020, the FDA has announced new enforcement priorities that are targeting the sale of flavoured vaping products as well as developing new requirements for premarket authorisation^20,21^. Revealing all the ingredient information would benefit numerous consumers who are susceptible to adverse reactions to flavours, such as contact dermatitis, and would allow clinicians to identify and prevent the right cause^22^.

There are a variety of programmes across the globe, which evaluate the safety of flavouring ingredients for use in food, such as the U.S. FDA, the European Food Safety Authority (EFSA), the Flavour and Extract Manufacturers Association (FEMA) and a Joint Food and Agriculture Organisation of the United Nations/WHO Expert Committee on Food Additives (JECFA)^23,24^. The FEMA Expert Panel constantly evaluates the safety of flavour ingredients, but only for their use in products that are ingested under the Food Additives Amendment^25^. Their generally recognised as safe (GRAS) status, generally a result of very small concentrations, does not corroborate with their use in vaping products (Fig. 1). As of 12 May 2020, recent action was taken in the United States to enforce manufacturers who wish to market flavoured vaping products to submit premarket applications to the FDA to demonstrate that the products are appropriate for the protection of public health.

In this work, we review how the concentrations of aroma compounds vary across the range of consumer products, with emphasis on e-cigarettes, where their amount is close to or even higher than it is deemed safe for consumption by the FEMA^23,25^. Some difficulty in predicting their final product concentration may arise due to their high volatility. However, this would not be an issue in e-cigarettes where the base product is PG and/or VG in which the flavourings are well solubilised and retained in a closed system.

A number of e-cigarettes, or vaping-associated lung injuries (EVALI), have been the focus of recent debate with regard to the safety of vaping products. In the vast majority of cases, it was suggested that the presence of tetrahydrocannabinol, the psychoactive compound in marijuana, or potentially vitamin E acetate, medium-chain triglyceride oil and other co-ingredients used alongside the vaping formulations, is the primary culprit in the development of EVALI^23^. However, there has not been enough time to assess any long-term use of vaping, particularly flavoured ENNDS. The safety of aroma compounds through inhalation is still in its early stages and does not take into consideration previous research on flavour compounds as a dietary ingredient, especially aldehydes and ketones, already known to interact with different proteins since 1970s first studies of food protein-flavour binding^26,27^. The novelty of this study therefore lies in the incorporation multiple aspects of flavour interactions in our daily life, aiming to draw attention to possible links between the elevated concentrations found in e-cigarettes, and any long-term VALI^28–30^. The resulting analysis is split into sections: toxicity of aroma compounds, final product concentrations, reports of cytotoxicity in flavoured e-cigarettes, impact on respiratory health and interactions of flavour compounds with various biological structures.

### Toxicity levels

Flavouring ingredients are deemed as safe for ingestion, provided they are present in concentrations below their toxic concentration, frequently denoted by the no-observed-adverse-effect level (NOAEL). However, all aroma compounds are inherently toxic at higher concentrations, having designated H (hazard statement) and P (precautionary statement) numbers, most of which are being used as organic solvents. Not surprisingly, the FEMA and the EFSA reject any claims that the use of GRAS flavours through inhalation are safe, stating that the flavourings are not evaluated for use in products other than ingested human food^23,31^. However, current regulations do not prevent vaping businesses for misusing any flavour ingredients listed as GRAS. In this report, most published monographs on their toxicological evaluation by inhalation, such as the LD_50_ (lethal dose for 50% of the test population), are usually obtained from murine studies^32^. Some examples of their toxicity evaluation reports are listed below.

#### Diacetyl

Diacetyl is a key compound in the flavour of butter, also produced naturally during the early stages of yeast fermentation. Diacetyl is added to margarine, alongside beta carotene that imparts yellow colours, in order to mimic some of the butter-like characteristics. It is also added to beverages, such as dark ales or other products, which have creamy flavours. However, in the early 2000, it was found that diacetyl is the cause of bronchiolitis obliterans (obstruction of bronchioles due to inflammation), if high concentrations are inhaled^33^. As a result, the use of diacetyl has now been banned in a range of products^34^. Toxic doses for diacetyl remain controversial, but in murine trials, it was found that exposure to over 100 ppm caused necrotising rhinitis, necrotising laryngitis, bronchitis and even death after exposure for only a few hours^33,35^.

#### Fruity esters

Esters are fundamental to the aroma of ripe fruit, such as apple and strawberries. Ethyl acetate, ethyl butyrate and ethyl hexanoate are amongst the most abundant esters, present in virtually every fruity product. High concentrations of ethyl acetate, ethyl butyrate and ethyl hexanoate are also used in perfumery. Isoamyl acetate is another commercially important ester, produced from acetic acid and isoamyl alcohol, and is used as a traditional banana flavouring. Naturally, the compound is found in bananas, but can also be found as a fermentation by-product. Isoamyl acetate is also released by honey bee’s stingers, which helps to attract and provoke other bees to attack^36^. Generally, fruity esters are considered to have low toxicity; however, according to the Center for Disease Control and Prevention (CDC), they can irritate the mucosal surfaces if inhaled, and were shown to cause skin damage at concentrations above 50–400 ppm, and fatigue, respiratory irritation and dyspnoea at concentrations over 1000 ppm^37^.

#### Benzaldehyde and cinnamaldehyde

The main ingredients in almond and cherry flavour in confectionary and soft drinks, are also found in the essential oil in almonds, apricots, cheery, seeds of peaches and laurel leaves: the ingestion of benzaldehyde appears to have relatively low toxicity; however, eye and inhalation exposure in rats to over 500 ppm was shown to have clinical symptoms of skin sensitisation, intoxication and delayed body weight gain. Mild exposure to low concentrations caused oedema, erythrema and nasal irritation in rabbits, while exposure to over 750–1000 ppm was found to result in death^38^.

Cinnamaldehyde is what gives cinnamon its distinctive flavour, and is used in multiple food and cosmetic products, such as chewing gum, ice cream, beverages and e-cigarettes in concentrations of up to 5000 ppm. It is also used in household products as a mosquito repellent active from concentrations as low as 29 ppm^39^. It is naturally produced by distillation of cinnamon tree bark, which contains about 2% pure cinnamaldehyde^40^. Ingestion is widely regarded as non-toxic at low concentrations, with cinnamic acid being the primary metabolite excreted in urine. However, its inhalation toxicity is limited to 125–800 mM (100 mg/ml or 100,000 ppm), which was reported to result in severe irritation of respiratory airways and coughing (TOX/2019/25).

#### Vanillin and ethyl vanillin

Most of the market for vanillin and ethyl vanillin are used by chocolate and ice cream manufacturers^41^. They are also used in numerous household products as a scented chemical used to counteract unpleasant odour. Due to their high odour threshold, concentrations of up to 1000 ppm are common^41^. Skin sensitisation has been reported in humans, and some studies reported site-specific damage to the liver, kidneys and spleen in rats at concentrations over 10,000 ppm, but is widely considered to be of low toxicity^42^. However, it has been found to be involved in a number of interactions with DNA^43,44^.

#### Limonene

It is a key ingredient of citrus flavour, found in all citrus fruit, mainly produced by extraction and distillation of oil-pressed by-products from the citrus fruit industries. It is not only used in food and other products, but has recently found applications in 3D printing, due to its ability to dissolve certain types of plastics, used as scaffolding for the printed structures^45^. Because limonene is a reactive compound, it is also used in the synthesis of other terpene compounds. Safety evaluation for limonene established no serious risk associated with ingestion in rodents, and NOAEL up to 250–500 mg/kg/d^46^. Inhalation exposure to low concentrations was shown to result in no signs of irritation or effects on the central nervous system (CNS), though it was concluded that limonene accumulates in the adipose tissue^47^. It was also recently identified that limonene and linalool oxidise under aqueous conditions, and their products can elicit allergic reactions and skin sensitisation^22^.

#### Maltol and ethyl maltol

They are common aroma compounds used in confectioneries for their caramel, candyfloss and other caramelised sugar-like flavours. They are also present in the aroma of freshly baked bread and is therefore used as a flavour enhancer in breads and other bakeries. Maltols are widely used in e-liquids at concentrations of up to 5% or more, adding a distinctive sweet candyfloss flavour^48^. Ethyl maltol is less toxic than maltol, which showed signs of growth inhibition and kidney damage in rats, at a concentration over 1000 mg/kg/d^49^. Maltol and ethyl maltol are also used as an alternative excipient for the delivery of iron, as ferric maltol complexes, in which three maltol monomers stabilise one ferric ion^50^. Commercial products such as Ferracru are made into hard capsules containing 30 mg of ferric iron, although the concentrations of maltol within the composition were not found in the current literature review.

#### Methyl anthranilate

An aroma-active compound in the flavour of black grapes, jasmine, lemon, orange and strawberries, methyl anthranilate can be found in a range of products from confectionery, soft drinks, children’s medicine as well as e-liquids. It is often combined with ethyl butyrate and ethyl acetate to give a strong bubblegum, apple or grape flavour. Other uses include pesticides due to its ability to act as a bird repellent, as well as a honey bee repellent^51^. Safety data sheets on methyl anthranilate report serious eye irritation and recommendations to avoid inhalation with 2.24 ppm shown to cause rabbit skin irritation^52^. In addition, a NOAEL ingestion dose over 500 mg/kg/d was shown to result in acute toxicity, while ingestion over 10,000 mg/kg was shown to result in liver and kidney damage^53^. However, the compound can be found at high concentrations in e-liquids as high as 1000 ppm, at which the growth of *Escherichia coli* was shown to be inhibited^54^.

#### Menthol

An abundant flavour, imparting the characteristic cooling sensation, menthol is perhaps the most widely use aroma compound also shown to be antibacterial, anaesthetic, counterirritant as well as an opioid-receptor agonist^55^. The cooling effect is largely due to its ability to trigger the TRPM8 receptors responsible for the cooling sensation^56^. It is naturally produced from wild mint (*Mentha arvensis*), peppermint and other mint species where it occurs along menthone, which has a ketone group instead of a secondary alcohol group, making it slightly sweeter than menthol. Menthol is known to have very low irritating properties to the skin or mucosa; therefore, it is used in very high concentrations. However, continuous exposure to elevated levels of menthol was shown to cause severe systemic symptoms in mice due to its interactions with the TRPM8 receptors, which act as calcium ion channels not only on the olfactory bulb but also in other organs of the body^57^.

### Product concentrations

The concentrations of aroma compounds present in nature are extremely low (<1 ppm or mg/kg) and generally have a low odour detection threshold, which is the concentration at which a compound can be detected via orthonasal or retronasal olfaction. However, there is great variation, especially in foods that have undergone thermal treatment (e.g., coffee), or fermentation (e.g., cocoa, tea and beer), or in products that have intentionally been enhanced. Flavourings used in consumer products have a GRAS status and are approved for use in food and cosmetics^58^. However, their concentrations can significantly vary depending on the application. A summary of some of the reported concentrations in a range of consumer products have been listed in Table 1.

**Table 1:** Common aroma compounds and their concentrations reported in different consumer products (expressed as parts per million (ppm) where 1 ppm = 1 mg/l). The data were normalised to a single type of concentration units used in food analysis (parts per million—ppm, where 1 ppm is equivalent to 1.0 mg/l).

Concentration (ppm)	Natural sources	Processed food	Pharma and cosmetics	E-liquids
Diacetyl^106–108^	1.6–65.2	1.2–27000	–	–
Ethyl butyrate^109–111^	0.26–2	0.002–0.7	–	300–1100
Isoamyl acetate^112,113^	0.01–25	20–2700	30–500	230
Benzaldehyde^114–116^	0.02–2	~2–4	1–5000	21,000
Cinnamaldehyde^39,69,117^	0.01–31,500*	~122–311	200–4000	500–145,000
Limonene^110,111,118,119^	0.27–67	20–278	1–10	779–106,479
Ethyl acetate^110,120^	0.01–0.58	50–100	<150	7100
Ethyl maltol^121–124^	0	1–142	5000	1190–61,230
Vanillin^121,125,126^	0.2–0.6	60–500	10–10,500	33,000
Ethyl vanillin^68,121,127^	0	50	16,090	5400
Methyl anthranilate^112,128–130^	<0.5	0.02–17.5	0.02–1000	1300
Menthol^131,132^	–	<6000	800–4500	57,000
Menthone^68,70,131^	14	1.47–590	590	900
α-pinene^59,110,118,133,134^	0.10–97	~1–1.7	3–12	640–4800

*Present in cinnamon oil; – not identified.

If more than one value were identified (from different products of the same category), the concentration values are given as a range. Natural sources: raw fruit and vegetables and unprocessed animal products; processed food: flavours that have either developed naturally (Maillard reaction or Strecker degradation) or that have been additionally included; E-liquids represent the liquid formulation found in e-cigarettes,either (refill or disposable)^48,70^.

The collected data show that the concentration of aroma compounds present in natural sources is much lower than in processed food and other products. Some compounds such as cinnamaldehyde are naturally present at very high concentration in the oils of cinnamon bark. Interestingly, α-pinene can also be found at high concentrations in milk, which is reported in certain populations of cows fed on alpine rangeland diets. Therefore, it is not recommended for cows to eat plants that contain high concentrations of pinene such as juniper or pine needles, as it was found to induce abortion particularly in the last trimester^59–61^. Murine studies have shown that α-pinene inhalation exposure to 400 ppm led to a decreased body mass, liver and kidney defects, especially in female rats^62^.

Besides plant essential oils, which naturally contain high amounts of aroma compounds, there is a clear trend showing that the concentration of food flavouring present in e-cigarettes can be over ten-thousand times higher (Table 1). Generally, the levels of aroma compounds in cosmetics were found not too high, perhaps due to a more rigorous toxicological evaluation of pharmaceuticals and cosmetics, i.e., allergies or skin sensitisation testing^63^. However, the concentrations present in vaping products are often close to or even in excess of their levels deemed safe for consumption by FEMA^25^. This reflects on the gap in the regulation of food flavour compounds administered by inhalation. Based on the latest review on 64 new flavouring updates analysed by the Expert Panel, the concentrations available for inhalation identified in e-liquids (Table 1) are higher than the average maximum use levels (ppm) found in food products for cinnamaldehyde, vanillin, maltol, pinene and close to the limit for ethyl acetate^25^.

### Flavour inhalation and impact on respiratory health

#### Use of flavour in e-cigarettes

**Fig. 2:**
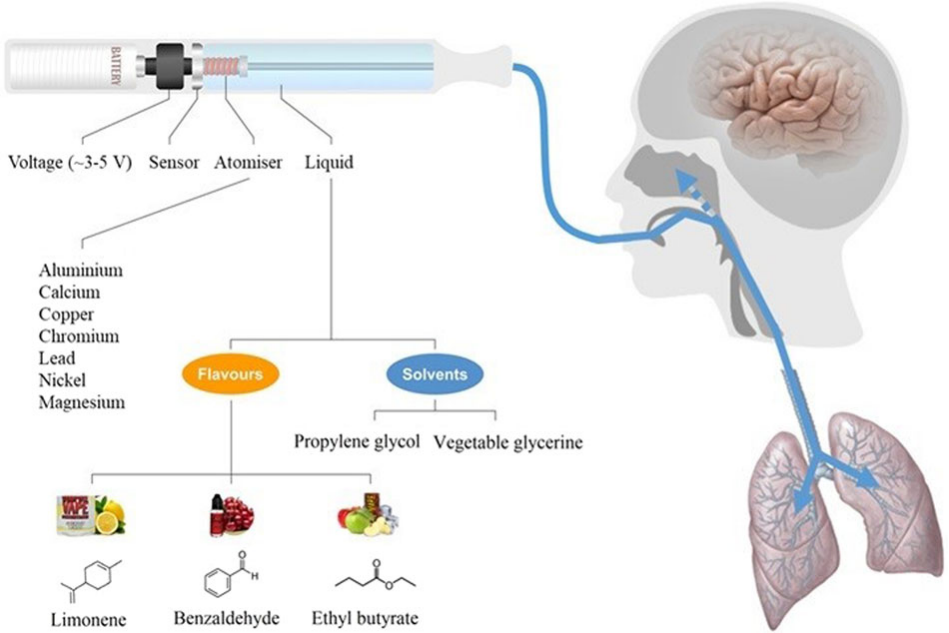
Structural representation of a typical configuration of a vape (ENNDS) device illustrating the mode of administration of flavour. The set-up consists of a battery, sensor, atomiser, coil and a refill tank. The blue arrow represents the aerosol generated, which is inhaled directly into the lungs. The perception of flavour is retronasal, predominantly given by the exhalation of the vapour.

In order to recapture the tobacco market, the makers of conventional cigarettes, along new SMEs have played a leading role in the fight against smoking and its consequences. This gave rise to the modern ENDS/ENNDS devices available to date. In order to encourage smokers to quit or at least switch to the healthier alternative, the industry aimed to increase the acceptability and enjoyment of ENDS by adopting some of the existing technologies available for the food industry. However today, the use of flavourings is more prevalent in the vaping industry than in any other. The flavour names are so attractive that not only they have successfully managed to encourage tobacco smokers to try them, but have also attracted a new and young population of vapers, which have never previously been exposed to nicotine products. In order to avoid this danger, and the efforts made to reduce the addiction to nicotine have led to the development of ENNDS, which simulates the physical action of conventional cigarettes, but without using any of the nicotine^64,65^. Their main selling point is simply the overwhelming variety of flavours and the overall social experience of vaping, partly dependent on the types of solvent (or bases) in which these flavours are dispersed in, usually a mixture of propylene glycol (PG) and/or vegetable glycerine (VG). The flavours, present at high concentrations, are vaporised by heating the liquid to high temperatures (depending on battery voltage) and inhaled straight to the lungs (Fig. 2). During vaping, the perception of flavour is retronasal given by the inhalation and exhalation of e-liquid vapours (Fig. 2). During these processes, a large part of the solvent vapours containing the different flavourings adhere to the mucosal membranes along the surface area of the respiratory system.

Unlike the food and vaping industry, the use of flavouring in conventional cigarettes is banned in most countries, apart from menthol that is still being used in some countries, so it is difficult to understand why they are approved for use in smoking’s safer alternative^66^. The main issue relates to their mode of absorption and interaction with the surface mucus during inhalation, since a wide range of compounds are skin irritants at higher levels, with unclear consequences in the respiratory system.

#### Concentration-related toxicity

The rapid increase in the number of flavoured e-cigarette users has created a potential public health crisis, which has started to be globally recognised. There has not been enough time to assess the impact of the prolonged inhalation exposure to aroma compounds, but it is highly likely that they are linked to systemic health effects starting from oral–nasal and respiratory tract impairments.

In 2012, one of the first studies looked at the cytotoxicity of vaping flavours on human pulmonary fibroblasts, human embryonic stem cells and mouse neural stem cells, and found that the effects are dependent on the type of flavour compound, with aldehydes like cinnamaldehyde showing the largest effect^67^. Later in Behar et al, (2014), it was found that cinnamaldehyde concentrations were as high as 40 mg/ml (4% or 40,000 ppm) in some cases, with other flavours also exceeding 5 mg/ml^68^. A more recent study published by the American Thoracic Society reported that cinnamaldehyde physically disrupts normal cilia physiology, affecting mucosal transport^69^. In 2016, Tierney et al. studied the concentrations of aroma compounds in 30 different products and found the concentrations of vanillin to be as high as 43 mg/ml, while concentrations of ethyl maltol, methol and benzaldehyde exceeded 20 mg/ml^70^.

#### Buttery and sweet compounds

Some progress was made on diacetyl and 2,3-pentanedione, which are deemed safe for ingestion by the FDA, but were shown to be the cause for *Bronchiolitis obliterans*, an irreversible disease that causes alveoli obstruction. This was commonly referred to as “popcorn lung” since it was discovered during a diacetyl spillage at a popcorn production plant, in which the workers were exposed to large amounts of diacetyl vapour^71,72^. However, other buttery diketone flavours, which are now in use, are suggested to be no different in terms of toxicity by inhalation^71,72^. The use of diacetyl has therefore been banned in inhalation products; however, one study found that almost 70% of e-liquids in 159 products still contained diacetyl^73^. The study by the National Institute for Occupational Safety and Health identified that over 40% of the refill fluids contained diacetyl and 2,3-pentanedione in concentrations exceeding the recommended standards^73^.

In some products, the use of sucrose has been added as an ingredient for its sweetness^74,75^. Heating sucrose solutions results in the productions of sweet aroma compounds, such as hydroxymethylfurfural (HMF) and furfural (FA) in concentrations that are dependent on the voltage and the type of heating elements used in the device. The exposure levels have been found to be similar to those in cigarette smoke^74^. Exposure to HMF concentrations as low as 2.6 mg/ml were found to be toxic, being linked with potential mutagenic activity in humans, while for FA, there are reports of carcinogenic activity and histopathological changes in the respiratory epithelium of mice^76^. In another study, exposure to vapours decreased the metabolic activity and cell viability, and increased the levels of interleukin (IL)-1β, IL-6, IL-10, CXCL1, CXCL2 and CXCL10 compared to controls, with strawberry-labelled flavour being the most cytotoxic^77^.

#### Carbonyl-containing compounds

Research continued to analyse the effects of flavourings and carbonyl-containing flavours such as aldehydes and ketones. In Qu et al., benzaldehyde was detected in 108 out of 148 ENDS tested, and was shown that flavoured ENDS emits the highest concentrations of aldehyde by-products, up to 38.1 times higher than unflavoured samples^78^.

Some carbonyl group compounds, such as ethyl acetate, have originally been used as solvents in glues or nail polish remover at high concentrations, with concentrations over 400 ppm of ethyl acetate posing serious risk for eye, nose and throat irritation, as well as drowsiness and weakness^79,80^. Animal studies showed that ethyl acetate affects the mucous membranes of the respiratory and conjunctiva with concentrations exceeding 20 000 ppm being linked to CNS effects, pulmonary oedema and haemorrhages (CDC, 2018). In the series of studies led by James F. Pankow and Prue Talbot, a number of products have been found to contain ethyl acetate ranging from 200 ppm to 7200 ppm, with concentrations over 10,000 ppm also reported^48,67,68^.

Aldehydes were found to represent the largest fraction, with vanillin and ethyl vanillin being at the top. Other compounds, such as cinnamaldehyde, benzaldehyde, ethyl maltol, ethyl acetate and ethyl butyrate, were also found to be close to or much higher than the recommended workplace exposure limits, based on the consumption rate reported on vaping forums^70^. In a follow-on study, Behar et al.^68^ analysed the concentrations of flavouring compounds in 39 commercial refill fluids, previously evaluated for cytotoxicity. Twelve dominant compounds were identified with 29% of the products at levels higher than 12,000 ppm, much higher than the median nicotine concentration. Most of them were found to be transferred very efficiently into the aerosols (mean transfer = 98%) with even higher concentrations being delivered at a higher battery voltage (3 and 5 V). Their cytotoxicity was used to create a library of refill fluid lexicon, such as creamy, minty, sweet, fruity, tobacco and spiced. It was found that the cytotoxicity data accurately predicted 74% of the aerosol flavours, with creamy or buttery flavours being the most cytotoxic. Moreover, diacetyl was still identified as a secondary-reaction product generated at higher voltage (5 V) in concentrations ranging from 20 to 100 ppm, particularly in samples containing cinnamaldehyde, benzyl alcohol, triacetin and acetoin^68^. In one particular sample, the concentration of cinnamaldehyde was 34%, corresponding to more than a 100,000 times its cytotoxic concentration^48^.

#### Cytotoxic effects of flavour on human cells

Omaiye et al. (2019) investigated 277 commercial refill fluids, and the most common flavour compounds were tested for cytotoxicity on human pulmonary fibroblasts and epithelial cells using the MTT assay kit. It was found that 85% of the samples had a flavour concentration higher than 1000 ppm, while 37% of them had concentrations exceeding 10,000 ppm (>1%), also reinforced by follow-up studies^81^. Ethyl maltol was present in over 80% of the samples, while menthol, cinnamaldehyde and triacetin were present in half of the samples. Although some compounds are being generally regarded as non-toxic, the cytotoxicity assay showed that menthol and ethyl maltol were found in over 100 times and 30 times higher than their cytotoxic concentration^48^.

Some studies even raised the question to whether the presence of flavours has any positive effect at all on the reduction of cigarette smokers as opposed to the use of unflavoured or menthol-only ENDS. One study was performed on 88 smokers who were given different types of flavoured ENDS to use as a substitute for smoking over a 6-week period. The analysis showed that the rate of cigarette smoking decreased significantly during this period from an average of 16–7 cigarettes a day. However, the only flavour, which contributed significantly to the decrease in smoking, was menthol, with the other flavours such as chocolate or cherry playing an insignificant role in the smoking cessation trial^64^. A later study also showed that non-flavoured vapes had a significantly lower cyctotoxicity compared to flavoured E-liquids^82^.

#### VALI and the respiratory microbiome

The CDC is a leading epidemiological organisation in the world. In 2019, it found that over 1400 respiratory cases linked to VALI in the United States, a term characterised by respiratory distress with bilateral (sometimes haemorrhagic) infiltrates within 3 months of using ENDS/ENNDS^83^. Aside from respiratory illness, recent studies also found that vaping can change the oral and lung microbiome, leading to implications in cavities, gum disease and other health issues^84^. The oral microbiome in participants who vape was found to contain significantly higher levels of *Porphyromonas* and *Veillonella* bacteria, the latter being found in cigarette smokers as well. The altered microbiome was found to contribute to a higher concentration of inflammatory response cytokines^84^. It also remains to be seen whether a history of VALI could pose higher risks for influenza and other viral lung infection complications^85^. As a result, the CDC, FDA and the Surgeon General of the United States urged state health departments to educate the public in how to lower the risk of viral infections by avoiding smoking or vaping, including exposure to second-hand aerosol^86^.

On a positive note, some aroma compounds found in plant essential oils, like eugenol, *β*-caryophyllene, limonene, geraniol, cineol, myrcene and cinnamaldehyde, are known to possess antimicrobial, antifungal and antiviral properties. The exact molecular mechanisms of interaction are not yet known; however, studies are beginning to highlight their important role in health treatment applications^87^. In addition, their use as expectorants, such as monoterpenes, has been globally recognised, provided they are prepared at the correct doses. For instance, double-blind, placebo- controlled trials confirmed that the essential oil administration is more effective than placebo, and helped in alleviating symptoms of chronic bronchitis and acute sinusitis^88^.

### Interactions of flavour compounds with biological structures

#### Flavour interactions in aqueous media

**Fig. 3:**
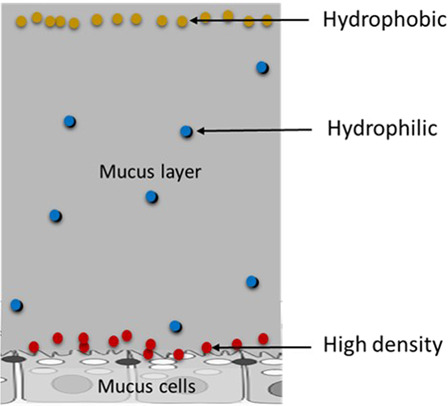
Distribution of aroma compounds in aqueous media. Schematic representation of the protective mucous layer showing the possible distributions of flavour molecules, depending on their physical and chemical properties.

Irrespective of the mode of administration of flavour compounds (ingestion or inhalation), their behaviour on the intestinal or respiratory mucosae will be determined by the same type of interaction mechanism. Interactions with aroma compounds are generally classified into three categories^89^: (i) binding of flavour compounds, (ii) phase partitioning, i.e., air, water or lipid or (iii) viscosity effects. Binding can either be reversible or irreversible depending on the strength of the interaction. Ketones and aldehydes are reported to covalently bond with the amino groups of proteins^27,90,91^. Others have been shown to form weak hydrogen bonds with food or salivary macromolecules containing electronegative clusters of nitrogen, sulfur or oxygen^92^. Thermodynamic and kinetic mechanisms of interactions involving aroma compounds depend on the physical and chemical properties of each individual compound^3^. Most of them are lipophilic with very small-chain compounds such as acetaldehyde being highly soluble in water as well (Fig. 3). In addition, each molecule has a different freezing and boiling point that would have a significant effect on the properties of the base in which they are dissolved in, such as density and viscosity, but also on the properties of the biological system involved in which the flavour is present (i.e., saliva, mucus and cell membrane proteins). While low concentrations of aroma compounds may have negligible physical and chemical effects, the very high concentrations of flavourings would contribute significantly to the physical and chemical properties of the biological system, including viscosity effects and interaction with proteins, as will be discussed in the next section.

#### Flavour protein interactions

Mucin glycoproteins are the predominant constituents in the mucus, present in different sizes and shapes across the gastrointestinal tract, but also within the lining of lungs. They have the formidable ability to act as a permeability barrier, regulating access across to the epithelium. Most mucins are heavily glycosylated, although they do contain regions of unglycosylated polypeptide, predominantly composed of cysteine, serine, threonine and proline^93^. Serine, threonine and proline assist in the coiling of the glycoprotein, which may give rise to potential protein-aroma interaction sites through hydrogen bonding and hydrophobic interactions. As reported with food proteins, it is hypothesised that the amino groups, present on the surface of mucus proteins, can interact with the aldehyde and ketone flavourings, and lead to the formation of permanent or non-permanent adducts that can lead to impaired mucosal function and other physiological disorders once they pass into the bloodstream.

Such interactions have been reported for the shorter more hydrophilic aldehyde—acetaldehyde— which is linked to the onset of mutagenesis, DNA damage, heart failure and coagulation disorders^94^. Particularly associated with chronic alcohol consumers, acetaldehyde was shown to increase the risk of developing alcoholic liver disease and hepatocellular carcinoma through protein adduct formations, leading to non-permanent but also permanent impairment in protein function^95^.

Aroma-protein interactions have also been reported for more complex molecules. Interactions with longer-chain aldehydes, such as pentanal, hexanal and up to decanal, have been reported in the presence of saliva and other protein structures, reinforcing the hypothesis of Schiff bases in aqueous solutions observed in vitro in recent studies^96^. In other studies, vanillin and ethyl vanillin, which also contain additional hydroxyl and ether groups, were reported to interact with DNA at the A–T groove region under physiological conditions^44,97^. Other work also found the use of vanillin as a DNA-dependent protein kinase inhibitor for use in anticancer therapies^44^.

Interactions with phenolic aroma compounds have also been documented. For instance, *p*-cresol, a by-product of citral degradation, which is present in some citrus-flavoured drinks, was shown to interact with mucins to result in the formation of smaller fragments^93^. A similar compound is *m*-cresol that has long been used as an excipient in insulin formulations in order to de-aggregate insulins and keep the proteins in their active, monomeric form^98^. Guaiacol is another related compound that is a substituted phenol used in the synthesis of other aroma compounds, but also naturally present in the flavour of whiskey. It is used as a universal substrate for peroxidase enzymes, through the interaction with glycine and isoleucine with its phenoxy group^99^. A similar mechanism is reported for the mucilatory clearance effects of guaiacol glyceryl ether—or guaifenesin, administered to patients for thinning of the mucus^100^. Cinnamon is also known for supressing α-amylase activity, the most abundant salivary and pancreatic enzyme, therefore investigated for its anti-hyperglycaemic properties. As a result, cinnamaldehyde has been proposed for use in diabetic intervention therapies^101^.

#### Flavour perception outside the olfactory bulb

Recent studies have also shown that aroma compounds play a role in cell signalling outside the olfactory region^57,102^. The olfactory receptors are a class of chemosensory G-protein-coupled receptors (csGPCRs), which are guanine nucleotide-binding proteins acting as molecular switches inside cells, by translating stimuli into chemical signals inside the cell^57^. They are the largest groups of GPCRs, that we use on a daily basis to distinguish food flavour and other environmental stimuli. However, what is less known is that these receptors are not only present in the olfactory bulb, but also expressed in other internal tissues and organs that are not related to the detection of odours^102^. It remains unknown whether these low-molecular-weight flavour compounds interact with the receptors, not for olfaction, but for affecting or regulating the internal environments in their presence. Based on these findings, it was therefore hypothesised that csGPCRs have a high potential for pharmacological relevance as drug targets, since nonetheless the majority (35%) of approved drugs act by modulating 7 transmembrane (7TM) receptors, which are GPCRs^57^. At the moment, the number of GPCRs targeted by drugs represents ~16% of the ~800 GPCRs identified in the human genome^103,104^. It is therefore worth noting the potential physiological involvement of flavour compounds in other tissues and organs that might provide clues to the onset of serious diseases, like cancer, metabolic and respiratory diseases^105^. Yet, it remains unknown what is the likelihood of interaction and the effect of aroma compounds diffusing through the mucosal lining into the blood, and the likelihood of interactions with the csGPCRs in other organs. As proven by evolution, flavour compounds in our environment may have either beneficial or harmful effects; therefore, it is imperative to examine the effects at the high concentrations present in products such as e-cigarettes. It might be that the effects are subtle in the short term, as seen with the other ingredients (fat, sugar and salt), but enough to fuel a new generation of serious NCDs in the long term.

### Further remarks

In the battle to provide healthier diets and lifestyles, continuous efforts are needed to ensure that the quality and quantity of food ingredients are well regulated and made accessible to the consumer. However, in most products, including food, household, pharmaceutics and flavoured e-cigarettes, the type and concentration of flavour compounds are not disclosed. This is timely for flavoured e-cigarettes, in which food flavouring represents their only ingredient. Currently, the high concentrations of pure compounds in e-cigarettes are identified as close to as or even higher than the safety standards given by their GRAS status. This is starting to be globally recognised, and while safety evaluations are continuously updated by experts, such as the Expert Panel of the FEMA, research is needed to identify and quantify these interactions in terms of the oral, respiratory and intestinal effects associated with a high flavour intake. In this regard, the toxicity of flavour chemicals should be re-evaluated, particularly with regard to inhalation, but also ingestion, both of which are not well understood, at least not in the long term. While studies have demonstrated cytotoxic effects of flavour at the cellular level, very few have yet focused on the interactions with the mucus, which acts as the permeability barrier regulating the transfer of gases, liquids and nutrients across the mucosal membrane.
